# Erika Love (1925–2020)

**DOI:** 10.5195/jmla.2021.1240

**Published:** 2021-07-01

**Authors:** Gale G. Hannigan, Ana D. Cleveland, Jonathan D. Eldredge

**Affiliations:** 1 ghannigan@salud.unm.edu, Research Professor, Health Sciences Library and Informatics Center, University of New Mexico, Albuquerque, NM; 2 ana.cleveland@unt.edu, Regents Professor, College of Information, Department of Information Science, University of North Texas, Denton, TX; 3 jeldredge@salud.unm.edu, Professor, Health Sciences Library and Informatics Center & College of Population Health, University of New Mexico, Albuquerque, NM

## Abstract

Erika Love, MLA president and early advocate for research in libraries, died October 8, 2020. Erika held many leadership positions in the profession and received several MLA awards recognizing her contributions. She has been called “the midwife” of MLA's early research initiatives [1].

Erika Love, Medical Library Association (MLA) president and early advocate for research in libraries, died October 8, 2020, at the age of 95. Born in Germany, Erika grew up in an educated and culturally rich environment. Her family did not support the National Socialism regime and suffered political persecution and economic hardship. After World War II, she matriculated at the University of Heidelberg. She worked as a library assistant at Amerika Haus in Darmstadt, a postwar organization fostering cultural interchange. In 1948, Erika married Victor Lamar Love; they moved to Indiana, his home state [2].

## A LEADER IN MEDICAL LIBRARIES

At Indiana University, Erika earned her BA (cum laude, 1950) and her MA in library science (1953). As a student, she worked at the Indianapolis Public Library and Indiana University School of Law Library. In her first professional position, Erika established a medical library for a psychiatric training, research, and treatment facility at the Larue D. Carter Memorial Hospital in Indianapolis. By the time she left that position in 1967, she had developed an interlibrary loan network for the Indiana Department of Mental Health, created a summer internship program, directed the two libraries for that department, and established the library as a leader of its kind.

Erika's husband died in 1966. In 1967, she became the director of libraries at the Bowman Gray School of Medicine in Winston-Salem, North Carolina. There she increased staff, redesigned systems, revamped programs, and established an Allied Health Library.

In 1971, she was appointed deputy associate director, library operations at the National Library of Medicine (NLM). She assisted with the administrative portion of the NLM MEDLINE User Network, increasing the size to more than four hundred centers.

In an oral history interview, Erika said “she became homesick … for the academic community, the need to see a medical student walking through the library” [3]. In 1977, Erika became director of the Medical Center Library at the University of New Mexico (UNM) in Albuquerque, where she oversaw a move into a new building, initiated the development of the Native Health Database, and pioneered the library's outreach program while overseeing the transition to library automation and electronic collections.

**Figure:**
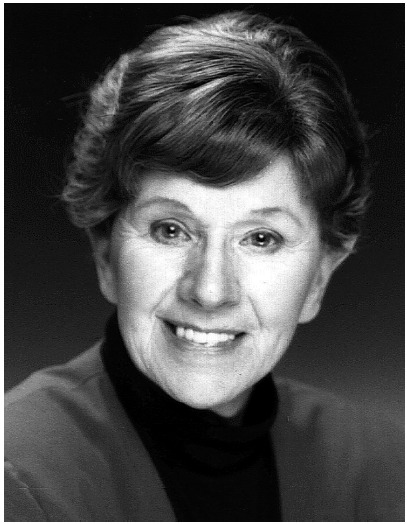


Erika understood the levers of administrative power. She knew where those levers were located and how to move them to benefit libraries. She recognized that those in charge of funding libraries spoke the language of accreditation, external grant funding, strategic planning, cost-effectiveness, and public opinion, so she went to great lengths to translate the utility of libraries in these terms. Her methodically prepared annual reports [4] and her newsletter, *adobe medicus* [5], from 1976–1996 at UNM reflect her efforts to merge the direction of the library with larger institutional priorities. To this end, she also cultivated close relationships with those above her. Manson Meads, the former dean of the Bowman-Gray Medical School where Erika worked in the 1970s, described having a “professional love affair” with Erika. She possessed both charm and intellectual prowess. At UNM, the director of the Medical Center, Leonard Napolitano, had a large external window in his office. Dr. Napolitano sometimes reportedly exited the back door of his office whenever he saw Erika approaching because he knew she wanted something, probably more funding for the library.

Throughout her career, Erika made many significant contributions to MLA. By the time she was awarded MLA's highest honor, the Marcia C. Noyes Award (1994), she had served in leadership positions in the MLA Midwest Regional Group and South Central Group, was MLA's representative to the Joint Committee on Hospital Libraries—Council of National Library Associations, directed the Mid-Atlantic Regional Medical Library, gave the Janet Doe Lecture, was a member of the MLA Board of Directors, an MLA Fellow, and served as MLA President (1978/79).

## A MENTOR AND ROLE MODEL (REMEMBRANCE BY JON ELDREDGE)

A recurring theme in Erika's career was her commitment to carefully recruiting and developing staff and the education and training of practicing librarians. Erika's UNM Medical Center Library attracted health sciences librarians from all corners of the nation during the 1970s and 1980s. When Erika first arrived in Albuquerque, I was working as a radio disc jockey and in a bookstore. I was so impressed with Erika and her associate director, Cecile Quintal, that I applied to the University of Michigan's School of Information. Erika recruited me to UNM five years later, where I served as an outreach librarian and later as chief of collections and information resources development. It was exciting to work with so many fascinating and capable colleagues. Erika was a forceful boss who expected her faculty and staff to give 100% to their jobs every day. She led by example and codified policies and procedures clearly outlining expectations of her faculty members. I attribute my career-long involvement in research to her encouragement.

Erika advanced the idea that library faculty members had to be true peers among their medical school counterparts through excellence in teaching and rigor in conducting applied research. In chairing the joint Task Force to Develop Guidelines for Academic Health Sciences Libraries, she “recast the role of the library as a partner … a support for the mission and goals of the parent institution” [1].

## AN ADVOCATE FOR RESEARCH AND A FRIEND (REMEMBRANCE BY ANA CLEVELAND)

Erika said “a profession that ignores the research component will become an appendage to other professions” [3]. As MLA president, Erika appointed an Ad Hoc Committee to Study MLA's Role in Library-Related Research. A result was the formation of the Research and Evaluation Committee and subsequently the Research Section.

It was in Lubbock, Texas, in 1977 at my first Annual Meeting of the South Central Chapter of the Medical Library Association that I met Erika. That was the beginning of our friendship. Her enthusiasm and passion for the profession and good humor radiated through the many moments we shared.

We moved within months of each other to the Southwest. I was starting my teaching career in Texas, and Erika was a new library director at UNM. Library education and research were at the center of our conversations. Erika's genuine interest in the education of medical librarians created a synergy between us that impacted my teaching. She had an incredibly analytical mind, and she made us question what we do. Of course, somehow, she found a research pathway to find answers and solutions.

As an educator, I welcomed her opinions about recruiting for the profession and the nature of our curriculum. She was also interested in the pedagogical methods used to deliver content. We compared library education and medical education and looked for similarities and differences.

We had lively dialogues about library education and the lack of robust research courses. After Erika's Janet Doe Lecture in 1987, “The Science of Medical Librarianship: Investing in the Future” [6], I recall having a heated discussion on the existence or lack of research courses in most of the library schools. She had made a reference to this in her lecture, and I was “a bit” defensive because research methods was a required course at my library school. We were having dinner and, by the end of the meal, we had come up with a plan on how to enhance the research skills of the members of the profession. We laughed at how passionate we both were about the topic!

Going to dinner with Erika was multifaceted. For me, it was a social event having an undercurrent of warmth and joyful conversation, and it also included a professional thread that raised more questions than answers. I remember clearly how she would tell me that I needed to build the research infrastructure in the curriculum, and over time, we would reap the benefits. Not only would we enrich the research knowledgebase of our profession but we would also create a workforce with research skills. There is no doubt that Erika had profound concern as to where graduates of library schools could get more education in conducting research. I have often thought how she would rejoice to know that MLA now has the Research Training Institute for the sole purpose to meet the needs that she often stated.

There was more than the professional bond between us. We both were careful to find balance in protecting our private lives and, at the same time, share with others our passion for the profession of medical librarianship and have our voices heard. It was those conversations with a personal overtone that had a special meaning. We showed interest in each other's backgrounds. We both immigrated to the US—Erika from Germany and I from Cuba. Both of our fathers were university professors, and both of our husbands had cancer. We shared stories, and the conversations could have gone on forever! The most fun was sharing our love of scarves, cooking, and traveling.

Over the years, I realized that, in her own way, she was mentoring me when I started my teaching career. I am grateful to have had her presence in my life.

## A COLLEAGUE WE WILL MISS

In medical libraries, Erika found a lifelong career. In turn, medical librarians found a leader, a colleague, an inspiration, and a friend. She said, “I never [told] people I was an information scientist … I've always been happy to say ‘I am a librarian’?” [3].
